# Endogenous 3-Iodothyronamine (T1AM) and Synthetic Thyronamine-Like Analog SG-2 Act as Novel Pleiotropic Neuroprotective Agents through the Modulation of SIRT6

**DOI:** 10.3390/molecules25051054

**Published:** 2020-02-26

**Authors:** Lorenza Bellusci, Massimiliano Runfola, Vittoria Carnicelli, Simona Sestito, Federica Fulceri, Filippo Santucci, Paola Lenzi, Francesco Fornai, Simona Rapposelli, Nicola Origlia, Riccardo Zucchi, Grazia Chiellini

**Affiliations:** 1 Laboratory of Biochemistry, Department of Pathology, University of Pisa, 56100 Pisa, Italy; lorenza.bellusci@student.unisi.it (L.B.); vittoria.carnicelli@unipi.it (V.C.); simona.sestito@for.unipi.it (S.S.); riccardo.zucchi@med.unipi.it (R.Z.); 2 Laboratory of Medicinal Chemistry, Department of Pharmacy, University of Pisa, 56126 Pisa, Italy; massimiliano.runfola@farm.unipi.it (M.R.); simona.rapposelli@unipi.it (S.R.); 3 Department of Clinical and Experimental Medicine, University of Pisa, 56100 Pisa, Italy; federica.fulceri@unipi.it; 4 Sant’Anna School of Advanced Studies, 56127 Pisa, Italy; f.santucci@santannapisa.it; 5 Unit of Human Anatomy, Department of Translational Research and New Technologies in Medicine and Surgery, University of Pisa, 56126 Pisa, Italy; paola.lenzi@med.unipi.it (P.L.); francesco.fornai@med.unipi.it (F.F.); 6 IRCCS Neuromed, 86077 Pozzilli (IS), Italy; 7 Interdepartmental Research Centre of Ageing Biology and Pathology, University of Pisa, 56126 Pisa, Italy; 8 National Research Council (CNR), Institute of Neuroscience, 56124 Pisa, Italy; nicola.origlia@in.cnr.it

**Keywords:** 3-iodothyronamine (T1AM), thyronamine-like analogs, multi-target directed ligand, neurodegeneration, Sirt6, autophagy, ubiquitine-proteasome, long term potentiation (LTP), mhAPP mouse model

## Abstract

3-iodothyronamine (T1AM) and the recently developed analog SG-2 are rapidly emerging as promising multi-target neuroprotective ligands able to reprogram lipid metabolism and to produce memory enhancement in mice. To elucidate the molecular mechanisms underlying the multi-target effects of these novel drug candidates, here we investigated whether the modulation of SIRT6, known to play a key role in reprogramming energy metabolism, might also drive the activation of clearing pathways, such as autophagy and ubiquitine-proteasome (UP), as further mechanisms against neurodegeneration. We show that both T1AM and SG-2 increase autophagy in U87MG cells by inducing the expression of SIRT6, which suppresses Akt activity thus leading to mTOR inhibition. This effect was concomitant with down-regulation of autophagy-related genes, including Hif1α, p53 and mTOR. Remarkably, when mTOR was inhibited a concomitant activation of autophagy and UP took place in U87MG cells. Since both compounds activate autophagy, which is known to sustain long term potentiation (LTP) in the entorhinal cortex (EC) and counteracting AD pathology, further electrophysiological studies were carried out in a transgenic mouse model of AD. We found that SG-2 was able to rescue LTP with an efficacy comparable to T1AM, further underlying its potential as a novel pleiotropic agent for neurodegenerative disorders treatment.

## 1. Introduction

Growing evidence suggests the presence of a strong association between obesity and neurodegeneration [1,2,3]. A number of pathways may translate the occurrence of obesity into abnormal brain structure and function [4]. When these pathways, such as the PI3K/Akt signaling, are altered brain damage related to neuronal oxidative stress occurs. This stressful condition recruits the endoplasmic reticulum (ER) and mitochondria, which are subcellular structures specifically altered following PI3K/Akt dysfunction ultimately producing neuronal loss [5]. 

Sirtuins belong to a highly conserved protein family of seven members (SIRT1–7). These proteins guard homeostasis by sensing the energy status and cope with energy needs. In fact, sirtuins finely tune the availability of specific cell components. At the same time, the activity of sirtuins mitigates a variety of steps involved in neurodegeneration. Among the seven mammalian sirtuins, SIRT6 plays an essential role in regulating metabolic homeostasis, stress resistance and lifespan [6,7]. In addition, SIRT6 is critical to maintain genomic stability in the brain and its loss leads to toxic Tau stabilization and phosphorylation [8]. Therefore, SIRT6 could be relevant in Alzheimer’s disease (AD) and age-related neurodegeneration. 

All these findings suggest that specific drugs acting on sirtuins level may exert broad therapeutic potential against interlinked age-related diseases, such as obesity and various neurodegenerative disorders (NDDs). 

Recently, evidence was provided that 3-iodothyronamine (T1AM), an endogenous component of the thyroid endocrine system [9,10], reprogrammed altered metabolism when exogenously administered to obese mice at pharmacological doses (10 and 25 mg/kg) [11]. This effect was based on the activation of SIRT6-mediated pathways [12], which in turn actively rescued fatty acid and glucose metabolism. We found that these effects led to a significant weight loss, which was independent from food consumption [11,12]. Notably, our group also provided the first evidence that when systemically administered to mice at submicromolar doses T1AM and recently developed thyronamine-like analogs were able to improve learning and memory [13]. In addition, in the same study in vitro experiments revealed the ability of these compounds to potently induce autophagy in human glioblastoma cell lines (U87MG) via inhibition of mTOR phosphorylation by the PI3K-AKT-mTOR pathway [13].

Taken together, these data indicate that further studies may be pursued to explore the potential of T1AM and synthetic thyronamine-like analogs to act as pleiotropic agents [14] for the treatment of interlinked diseases such as obesity and neurodegeneration.

**Figure 1:**
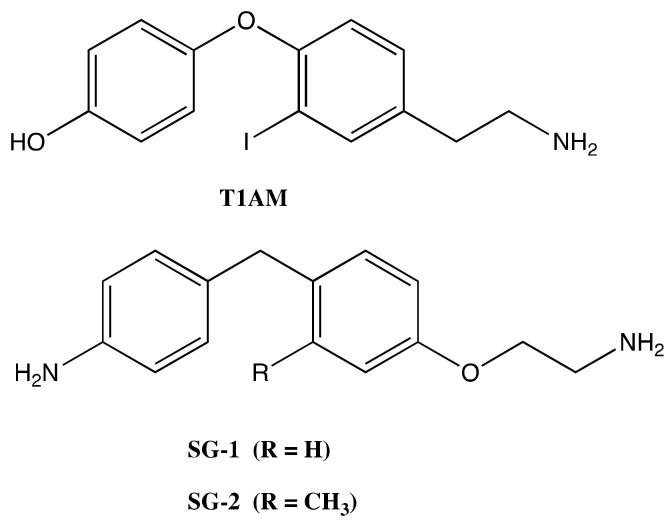
Structures of 3-iodothyronamine (T1AM) and thyronamine-like analogs SG-1 and SG-2.

Thus, the present work was designed to investigate in more detail the molecular mechanisms through which T1AM and recently developed thyronamine-like analogs (namely, SG-1 and SG-2, Figure 1), could produce a SIRT6 activation bridging autophagy, weight loss and neuroprotection [12,13].

The present investigation was further motivated by recent findings, which indicate that the two major clearing pathways, namely autophagy and ubiquitine proteasome (UP), considered as independent for long time, converge at the level of single organelles named autophagoproteasomes following mTOR inhibition. Such a merging when considered at functional level might empower protein homeostasis and cell clearing [15,16]. In line with this evidence, we included an experimental part where the merging between autophagy and UP was investigated under the effects of T1AM and thyronamine-like analogs SG-1 and SG-2.

In addition, previous studies have shown that administration of T1AM is able to rescue β-Amyloid induced neuronal dysfunction in wild type mice [17], and more recently, the protective effect of T1AM against neuronal plasticity impairment has been further confirmed in transgenic AD mice [18] (hAPP-J20, also denoted mhAPP mice), paving the way to the identification of new pharmacological targets to retard AD progression [19].

In this regard, with the aim to increase the therapeutic options for this incurable disease, we explored the ability of SG-2, already considered as a fully functional mimic of T1AM for behavioral and metabolic effects [13,20,21], to rescue β-amyloid neuronal dysfunction in mhAPP mice.

## 2. Results

### 2.1. Effects of SG-1, SG-2 and T1AM on the Expression of Autophagy-Related Genes

Autophagy is an important metabolic process which wraps unnecessary intracellular components, such as lipids, misfolded proteins, and dysfunctional organelles, in double-membraned vesicles, defined as autophagosomes, which are then transported to lysosomes for degradation and recycling, thus maintaining essential cellular viability in response to diverse stresses [22,23,24]. 

Previous studies have shown that mTOR can be inhibited by SIRT6 to promote autophagy in neuronal cells under oxidative stress [25]. Other groups have also demonstrated that SIRT6 induces autophagy by attenuating AKT/mTOR signal cascades in normal cells [26,27]. Above all, it seems that SIRT6 positively regulates cell autophagy by targeting mTOR signaling pathway. 

**Figure 2:**
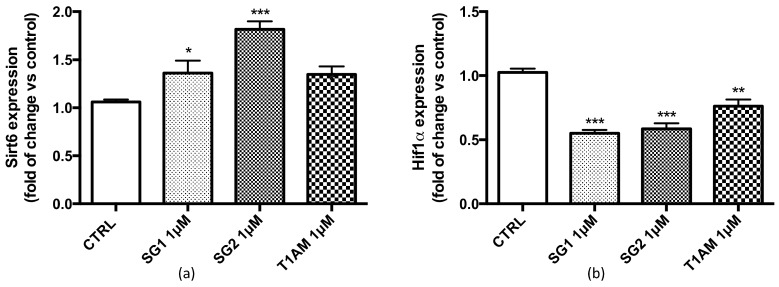

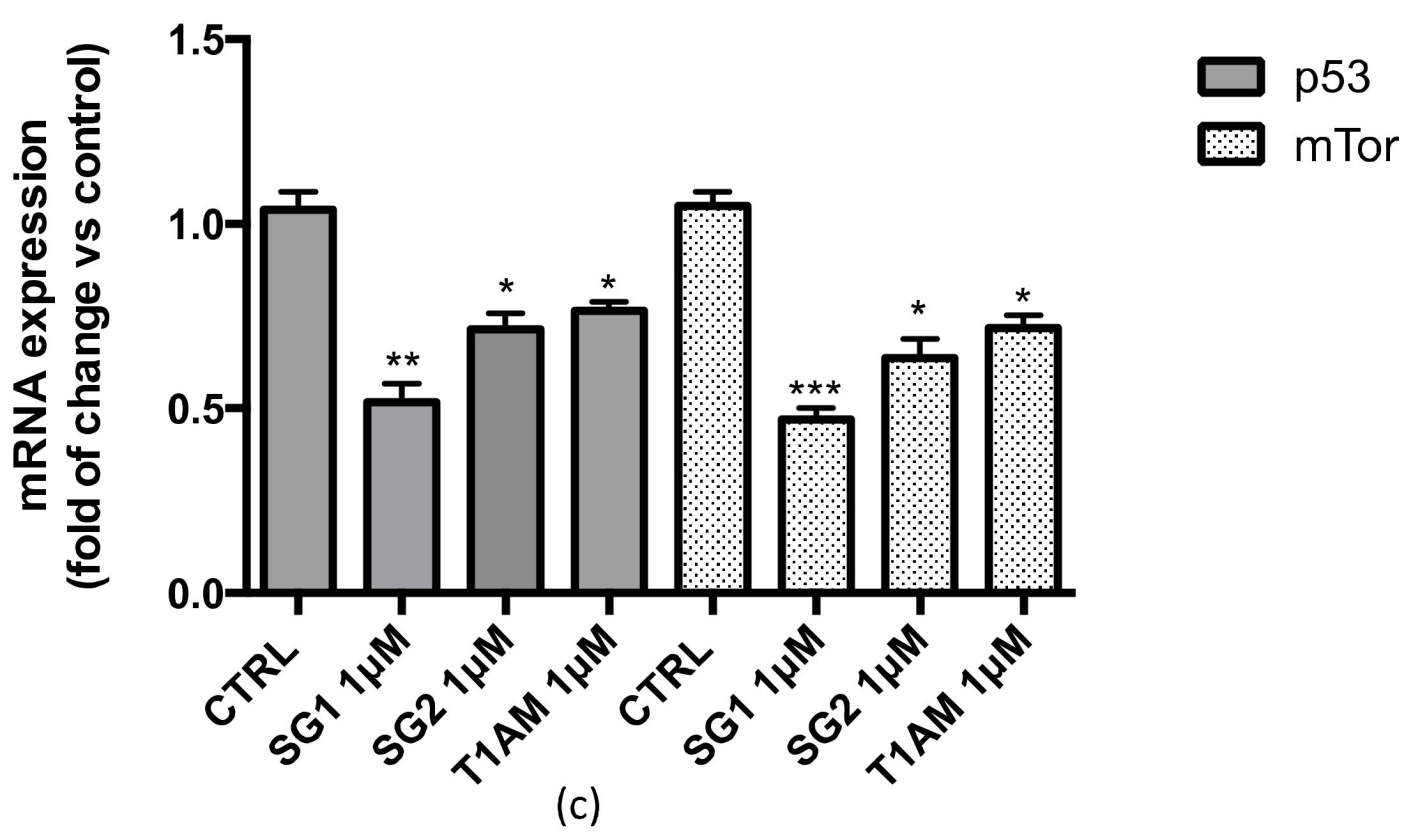
Effect of SG-1, SG-2 or T1AM treatment on the expression of autophagy ATG target genes in U87MG cells. After 24h incubation of U87MG cells with 1μM SG-1, SG-2 or T1AM total RNA was extracted, and the relative mRNA quantification of Sirt6 (**a**), Hif1α (**b**), or p53 and mTOR (**c**) was performed by real-time RT-PCR. The data are expressed as fold changes vs control and represent the mean ± SEM of three different experiments (*n* = 3), each performed in duplicate. Statistical significance was determined using a one-way ANOVA followed by a Dunnett’s post-test: * *P* ≤ 0.05, ** *P* ≤ 0.01, *** *P* ≤ 0.005 vs control.

In the present study, using the quantitative polymerase chain reaction assay (qPCR) we found that SIRT6 was highly upregulated in U87MG cells treated for 24 h with (1 μM) T1AM, SG-1 or SG-2 (Figure 2a). Recent studies [28] have demonstrated that SIRT6 regulates glucose homeostasis by inhibiting the hypoxia-inducible transcription factor (Hif1α), a key mediator of cellular adaptation to nutrient and oxygen stress. Our gene expression study indicated a reduced expression of Hif1α in U87MG cells after treatment with (1μM) T1AM, SG-1 or SG-2 (Figure 2b).

Known as a ‘guardian of the genome’ [29], p53 protein plays a crucial role in the development of neurodegenerative diseases, and high levels of p53 have been observed in the brain of AD, Parkinsons’s disease (PD) and Huntington’s disease patients [30]. Interestingly, inactivation of p53 by deletion, depletion or inhibition has been reported to trigger autophagy [30], and several distinct autophagy inducers, including rapamycin, stimulate the rapid degradation of p53 [31]. Here we tested the effects of SG-1, SG-2 and T1AM on the expression of p53 and mTOR in U87MG cells. Our results revealed a concomitant significant decrease of p53 and mTOR expression in U87MG cells after treatment with 1μM T1AM, SG-1 or SG-2 (Figure 2c).

### 2.2. Effects of SG-1, SG-2 and T1AM on the Expression of SIRT6 and mTOR 

**Figure 3:**
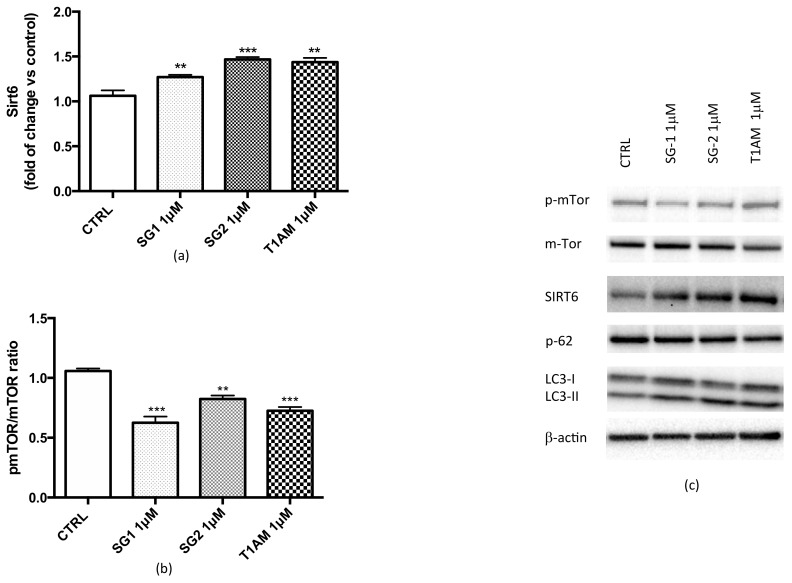
Western blot quantification of SIRT6 (**a**) and p-mTOR/mTOR (**b**) in U87MG cells exposed for 24 h to (1μM) SG-1, SG-2 or T1AM. β-actin was used as an internal control. Results represent the mean ± SEM of three different gels (*n* = 3). ** *P* ≤ 0.01, *** *P* ≤0.005 versus vehicle treated cells (CTRL). Representative western blots are shown in Panel **c**.

The transcriptional analysis results indicated that in U87MG cells a 24h treatment with 1μM T1AM, SG-1 or SG-2 induced a significant up-regulation of Sirt6 and a parallel down-regulation of mTOR. Protein expression studies by Western blotting confirmed over-expression of sirtuin 6 (SIRT6) (Figure 3a) and inhibition of mTOR phosphorylation (Figure 3b) in U87MG cell lysates (full length Western blots for Figure 3c are shown in Supplementary Figure S1). In agreement to our previous findings (13), increased level of LC3II, a marker for autophagosome formation, and decreased p62 protein level, a marker for autophagic protein degradation were also observed (Figure 3c), thus confirming the efficacy of T1AM and thyronamine-like analogs, SG-1 and SG-2, as autophagy inducers in U87MG cells.

**Figure 4:**
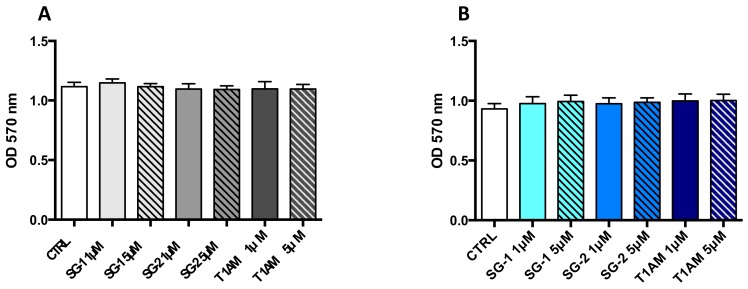
Compounds SG-1, SG-2 and T1AM do not promote toxicity in U87MG cells. Cell viability after 24 h (**A**) and 72h (**B**) of incubation with compound SG-1, SG-2 or T1AM. (1 and 5 μM) was assessed by MTT assay. The data represent the mean ± SEM of three independent experiments, each performed in triplicate (*n* = 3).

In addition, cellular viability was determined using the MTT colorimetric assay. No significant alterations of cell viability were observed in U87MG cells treated for 24 and 72 h with 1 and 5μM T1AM, SG-1 or SG-2 as compared to vehicle treated cells (0.1% DMSO) (Figure 4).

### 2.3. Effects of SG-1, SG-2 and T1AM on Cell Clearing Systems

As previously reported, T1AM and synthetic analogs SG-1 and SG-2 were found to produce a time-dependent recovery of autophagic activity in U87MG cells, due to the down-regulation of mTOR [13]. Recently, Lenzi et al. provided compelling morphological evidence that the two major clearing pathways of eukaryotic cells (autophagy and proteasome), converge at the level of single organelles named autophagoproteasomes [15]. At this level, the regulation of all components seems to rely on the status of mTOR activity. In fact, a dose dependent increase of these organelles was observed when the mTOR inhibitor rapamycin was administered [15]. 

**Figure 5:**
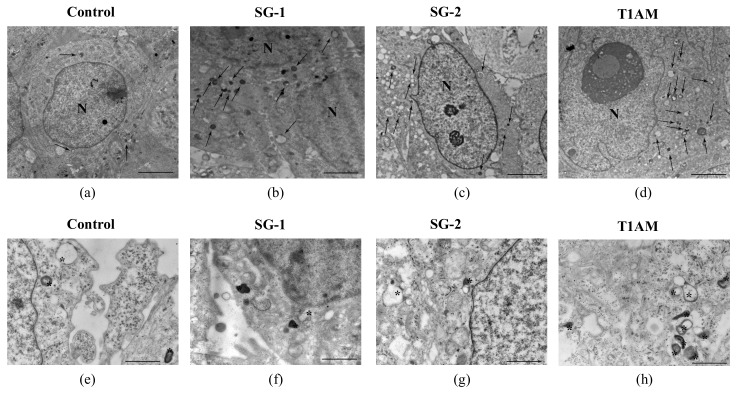

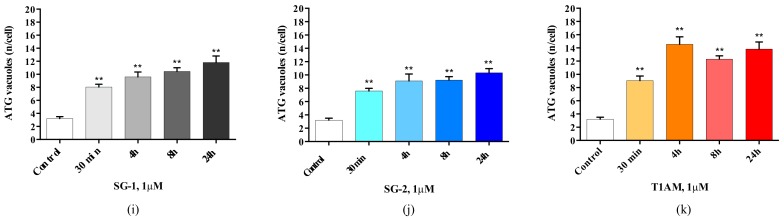
Transmission electron microscopy of U87MG cells. Representative pictures of -autophagy like vacuoles (arrows) in the cytoplasm in baseline conditions (**a**) and after treatment with 1 μM SG-1, SG-2 or T1AM at 24h, (**b–d**). High magnification of different shaped autophagy like vacuoles (asterisk) in baseline conditions (**e**) and after treatment with 1 μM SG-1, SG-2 or T1AM at 24h (**f–h**). The mean number of autophagy -like vacuoles per cell was counted and values were reported in graph for each compound (**i–k**). The graphs show that all compounds induce a time-dependent increase in the number of autophagy -like vacuoles compared with baseline conditions. Values represent the mean ± SEM of 30 cells for each group (*n* = 30). Comparisons between groups were made by using one-way ANOVA followed by a Dunnett’s post-test. ** *p ≤* 0.05 compared with baseline conditions. Scale bars (**a–d**) = 1µm; (**e–g**) = 160 nm.

In the present study, we tried to find out whether T1AM and synthetic analogs SG-1 and SG-2 have the ability to induce the occurrence of autophagoproteasome. Transmission electron microscopy (TEM) analysis confirmed a time dependent increase of autophagy vacuole density in U87MG cells exposed to the treatment with 1μM T1AM, SG-1 or SG-2, which generally leads to a roughly 3-fold increase as compared to U87MG cells in baseline conditions (Figure 5).

**Figure 6:**
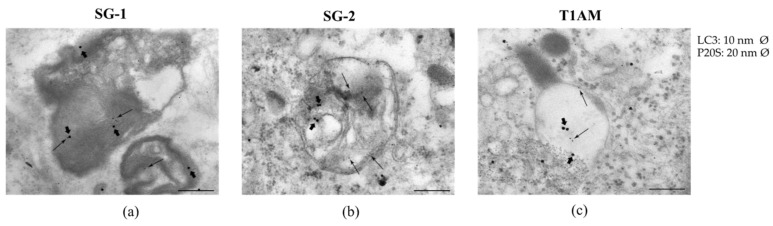

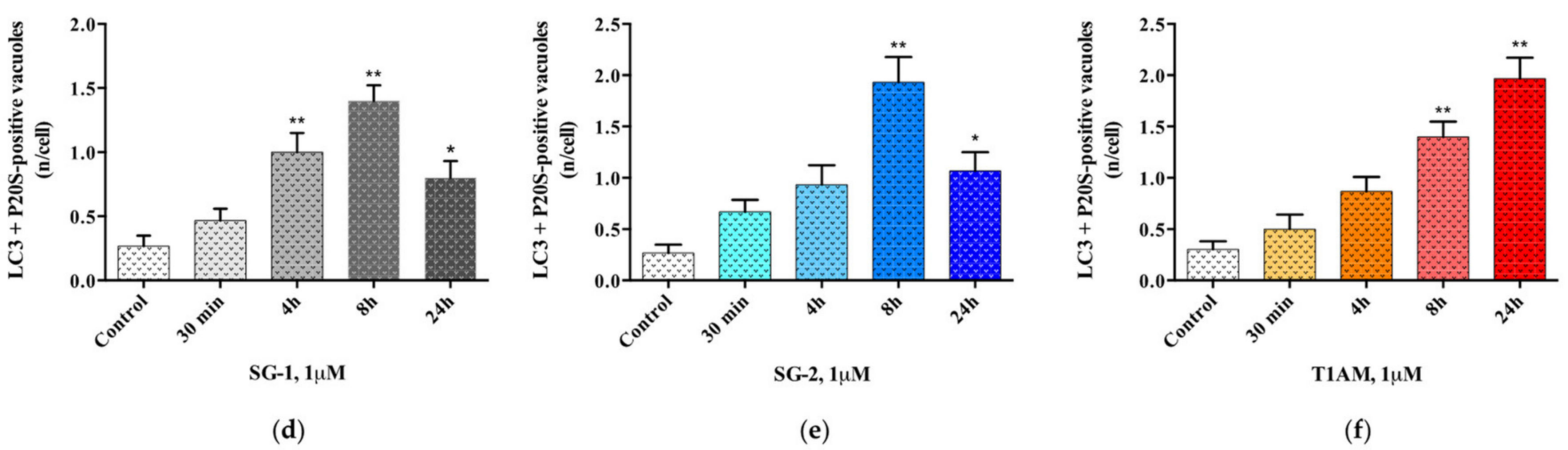
Occurrence of autophagoproteasome in U87MG cells after treatment with 1 μM SG-1, SG-2 or T1AM. As shown in representative micrographs (**a–c**), when LC3 and P20S immunogold staining was carried out concomitantly the occurrence of both P20S particles and LC3 particles within the very same autophagy vacuoles was documented in U87-MG cells exposed to 1 μM SG-1, SG-2 or T1AM. In these pictures P20S staining is represented by 20nm immunogold particles evidenced by arrowheads, while LC3 staining is represented by smaller (10 nm) immunogold particles evidenced by full thin arrows. After treatment with 1 μM SG-1, SG-2 and T1AM the mean number of autophagoproteasomes occurring in the cell was counted, and as shown in graphs (**d–f**), a time-dependent increase of P20S+LC3 positive vacuoles was documented. Values represent the mean ± SEM of 30 cells for each group (*n* = 30) Comparisons between groups were made by using one-way ANOVA followed by a Dunnett’s post-test. * *P* ≤ 0.05, ** *P* ≤ 0.01compared with baseline condition. Scale bars (**a–c**) = 50 nm.

As shown in representative panels (Figure 6), when LC3 and P20S immunogold staining was carried out concomitantly, the occurrence of both P20S and LC3 particles within the same organelle was documented in U87MG cells exposed to 1 μM SG-1, SG-2 or T1AM, suggesting that these compounds might modulate both autophagy and UP protein clearing pathways within the autophagoproteasomes. 

Interestingly, the time dependent increase in LC3 + P20S positive autophagoproteasomes observed in U-87MG cells under the effect of 1μM SG-1, SG-2 or T1AM (Figure 6) perfectly overlaps with the increase in LC3 positive autophagy vacuoles originally observed by Bellusci et al. in U87MG cells exposed to the same treatment [13]. Therefore, increasing mTOR inhibition seems to produce a quite harmonic expression of both clearing pathways.

### 2.4. SG-2 Rescues LTP in hAPP-J20 Mouse Model of AD at the Level of the EC

The evidence of pro-learning and antiamnestic effects elicited in mice by both T1AM and SG-2, along with their role in modulating autophagy, one of the potential therapeutic target processes in neurodegenerative diseases, encouraged us to pursue further investigations. Thus, we assessed the therapeutic potential of these molecules in the treatment of an experimental model of AD [32], a neurodegenerative condition which features early memory impairment. 

In this study, we used a transgenic mouse model of AD, which overexpresses alleles of the human APP gene (mhAPP mouse model) linked to familial AD [33]. These mice accumulate Aβ, beginning from entorhinal cortex (EC), which undergoes progressive neurodegeneration. Impairment of EC synaptic plasticity is an early sign of neuronal degeneration in mhAPP, which is evidenced by a loss of long term potentiation (LTP) expression [34]. 

Previous experiments showed that mhAPP slices, perfused with ACSF enriched with 5μM T1AM, regained LTP capacity after high frequency stimulation induction protocol (HFS) [18]. Notably, in a very recent work Accorroni et al. demonstrated that in the same experimental model co-administration of 5 nM EPPTB, a selective TAAR1 antagonist, was able to revert the rescuing effects of T1AM on LTP [35], suggesting a role of TAAR1 as a possible drug target to ameliorate cognitive dysfunction in AD patients. Since SG2 is a recently developed T1AM analog endowed of almost comparable TAAR1 agonist properties (EC50 SG-2 = 240 nM; EC50 T1AM = 189 nM) [36], we hypothesized that it may have a similar effect, and we decided to administer this compound in the perfusion medium at different concentrations. 

**Figure 7:**
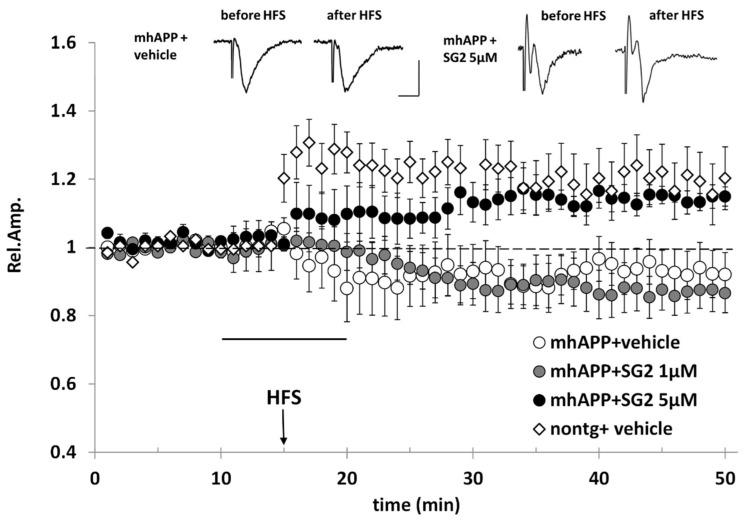
SG2 rescues LTP expression in the EC superficial layer of mhAPP mice. The LTP was induced by HFS stimulation in non-transgenic slices under ACSF (nontg + vehicle, white diamonds), whereas LTP expression was not elicited by HFS in mhAPP slices perfused with ACSF (mhAPP + vehicle, white circle) (*n* = 7). Perfusion of SG2 5μM (5μM, black circles, 10 min of application corresponding to the dark bar) rescued LTP expression in mhAPP slices (*n* = 6), whereas a lower concentration of SG2 (1μM, grey circles, 10 min of application corresponding to the dark bar) had no significant effect on mhAPP slices after HFS (*n* = 9). Error bars indicate SEM. Insert shows representative field potentials recorded either before or 35 min after HFS, in mhAPP slices under ACSF (mhAPP + vehicle, left) and in mhAPP slices under SG2 5μM (mhAPP+SG2 5μM, right). Calibration bars: 1 mV, 5 ms.

In agreement with previous evidence, our experiments showed that LTP cannot be elicited by HFS in mhAPP slices perfused with ACSF alone (Figure 7). We then tried to rescue LTP in mhAPP slices using the SG2 compound. However, when used at the lowest concentration (1µM), 10 min perfusion with SG2 was not effective, and the mean amplitude after HFS was not significantly different from that of mhAPP exposed to ACSF alone (0.87 ± 0.06, *n* = 7, versus 0.93 ± 0.044, *n* = 9). In contrast, a higher concentration of SG2 (5 μM), rescued LTP in mhAPP slices after HFS (1.14 ± 0.02, *n* = 6) to a level that was significantly higher than that observed in mhAPP slices alone (*p* < 0.05), as well as in mhAPP slices perfused with SG2 1 μM (*p* < 0.05) (Figure 7). 

## 3. Discussion

Neurodegenerative diseases are incurable multifactorial debilitating disorders of the nervous system that involve multiple pathways. Despite extensive efforts attempting to define the molecular mechanisms underlying neurodegeneration, many aspects of these pathologies remain elusive and no effective cures have been identified.

A common pathology shared by several neurodegenerative diseases is the accumulation of misfolded proteins. Given that autophagy is a cellular function that degrades abnormal proteins, including those that are misfolded, autophagy-inducing compounds are expected to mitigate the onset and progression of these diseases.

In the present study, we found that 3-iodothyronamine (T1AM), an endogenous component of the thyroid endocrine system originally identified as a potent trace amine associated receptor 1 (TAAR1) agonist, and recently developed thyronamine-like TAAR1 agonists, SG-1 and SG-2, increase autophagy in U87MG cells by inducing the expression of SIRT6 to suppress the activities of Akt and leading to the inhibition of mTOR activities, with SG-2 showing higher potency as compared to SG-1. Moreover, from our assays, increasing mTOR inhibition seems to produce a harmonic expression of autophagy and UP clearing pathways in U87MG cells. Even though the detailed mechanisms for SIRT6 in regulating autophagy and its interplay with the UP clearing pathway need to be explored in future studies, we can speculate that, through the modulation of Sirt6 expression, T1AM and its analogs SG-1 and SG-2 might exert pleiotropic effects on energy metabolism and cell clearing pathways, which is likely to sustain neuroprotection. 

Evidence that a 24h treatment of human glioblastoma U87MG cells with 1μM T1AM, SG-1 or SG-2, didn’t affect cell viability while increasing the expression of SIRT6, was provided. Notably, recent findings revealed that overexpression of SIRT6 can significantly affect glioblastoma cell growth and induce cell injury, resulting particularly evident 48 and 72h after cell seeding [36]. In addition, T1AM and SG-2 have been previously shown to reduce cancer cells growth when used at high micromolar concentrations [37]. In our study, all compounds under investigation, showed a lack of cytotoxic effect in U87MG cells when treatment was extended to 72h and the dose increased to 5μM. This observation suggests that, at least in our experimental setting, SIRT6 activation doesn’t cause human glioblastoma U87MG cells toxicity. Undeniably, future work will be required to ascertaining whether knocking down SIRT6 will thoroughly block out the autophagy induced by T1AM and SG-2 in U87MG cells and/or prevent the interplay between autophagy and UP protein degradation systems observed in the same cell line.

Our previous studies widely documented that SG-2 represents an effective functional mimic of T1AM with respect to TAAR1 agonistic activity [38], as well as to both metabolic and neurological effects [13,20,21]. In the present study, to further expand our knowledge on the pharmacological features of this novel T1AM analog (i.e. SG-2), we assessed the effects of SG-2 on the pathological alterations induced by Aβ in electrophysiological parameters, namely long term potentiation (LTP), in a transgenic mouse model of AD (mhAPP mouse) [32]. Indeed, the results obtained after testing the ability of SG-2 to restore LTP in mhAPP mouse showed that this molecule is also able to reproduce the protective effect of T1AM against Aβ toxicity at the level of the entorhinal cortex (EC). This finding may also strengthen the role of TAAR1 as a possible drug target to ameliorate cognitive dysfunction in AD patients [35]. As a further expansion of this preliminary study, since protein homeostasis is crucial to sustain synaptic long-term plasticity and counteracting AD pathology, in future studies we will direct our attention to evaluate whether T1AM and SG-2 are also able to produce SIRT6-dependent activation of autophagy in the EC of mhAPP mice. Indeed, a better understanding of the underlying mechanisms of neuroprotective and cognitive-enhancing properties of T1AM and SG-2 may have an impact in AD field and indicate the way to novel therapeutic approaches, possibly bridging autophagy, weight loss and neuroprotection.

## 4. Materials and Methods

### 4.1. Chemicals

3-Iodothyronamine (T1AM), was kindly provided by Prof. Thomas Scanlan (OHSU, Portland, OR, USA) and was dissolved in 0.5% DMSO (Veh). Thyronamine-like analogs SG-1 and SG-2, were synthesized by our group according a procedure previously described [38] and were dissolved in 0.5% DMSO (Veh) to perform experiments with U87MG cells. To carry out experiments with animals, aliquots of SG-2 were stored at −20 °C in DMSO as a 6.8 mM stock solution and diluted to the desired final concentration in artificial cerebrospinal fluid (ACSF), containing the following (in mM): 119 NaCl, 2.5 KCl, 2 CaCl_2_, 1.2 MgSO_4_, 1 NaH_2_PO_4_, 6.2 NaHCO_3_, 10 HEPES, 11 glucose.

### 4.2. Cell Lines

U87MG human glioma cell lines from Cell Bank (IRCC San Martino-IST, Genova, Itlay) were cultured in standard DMEM High Glucose medium (Sigma–Aldrich S.r.l., Milan, Italy) supplemented with 10% fetal bovine serum (FBS), penicillin (50 IU/mL) and 100 mg streptomycin (Sigma–Aldrich S.r.l.). Cells were kept at 37 °C in a humidified atmosphere with 5% CO_2_ and the medium was renewed two to three times per week.

For transmission electron microscopy (TEM), transcriptional analysis (qPCR) and Western blot (WB) assays, the cells were cultured at a density of 3 × 10^5^ cells/well in a 6-well plate in a final volume of 2 mL/well. 

Twenty-four hours after seeding, the cells were treated with test compounds (T1AM, SG1, and SG2) at the dose of 1 μM for different exposure times (0.5, 4, 8, and 24 h). Dilutions of test compounds were obtained by a stock solution (1 mM in saline containing 10% DMSO).

### 4.3. Assessment of Autophagy-Like Vacuoles, and Count of LC3 and P20S Immunogold Particles

U87MG cells were fixed with a solution containing 2.0% paraformaldehyde and 0.1% glutaraldehyde in 0.1 M PBS (pH 7.4) for 90 min at 4 °C. After removal of the fixing solution specimens were post-fixed in 1% OsO_4_ for 1h at 4 °C, they were dehydrated in ethanol and finally embedded in epoxy-resin. For ultrastructural morphometry, grids containing non-serial ultrathin sections (90 nm thick) were examined at TEM, at a magnification of 8000×. Several grids were analyzed in order to count a total number of 30 cells for each experimental group. We counted the number of autophagy vacuoles per cell as vacuoles with single, double or multiple membranes possessing the same electron density of the surrounding cytoplasm, or partly containing some electron dense structure [39]. In each cell we counted the number of autophagoproteasomes per cell as a single, double, multiple membrane vacuoles, in which immune-gold particles of LC3 (10 nm) and P20S (20 nm) were co-localized [39].

Post-embedding procedure was carried out on ultrathin sections collected on nickel grids, which were incubated on droplets of aqueous sodium metaperiodate (NaIO_4_), for 30 min, at room temperature in order to remove OsO_4_. Then grids were washed in PBS and incubated in a blocking solution containing 10% goat serum and 0.2% saponin for 20 min, at room temperature. Grids were then incubated with the primary antibody solution containing both rabbit anti-LC3 (Abcam, Cambridge, UK, diluted 1:50) and mouse anti-P20S (Abcam, Cambridge, UK, diluted 1:20), 0.2% saponin and 1% goat serum in a humidified chamber overnight, at 4 °C. After washing in PBS, grids were incubated in the secondary antibodies conjugated with gold particles (10 nm mean diameter, for gold particles anti-rabbit; 20 nm mean diameter, for gold particles anti-mouse, BB International) diluted 1:20 in PBS containing 0.2% saponin and 1% goat serum for 1 h, at room temperature. In control sections, the primary antibody was not applied and they were incubated with the secondary antibody only. After washing in PBS, grids were incubated on droplets of 1% glutaraldehyde for 3 min; additional extensive washing of grids on droplets of distilled water was carried out to remove extensive salt traces and prevent precipitation of uranyl acetate. Finally, the grids were counterstained with a saturated solution in distilled water of uranyl acetate and lead citrate and observed by using a JEM SX100 electron-microscope (JEOL, Tokyo, Japan).

### 4.4. Gene Expression Analysis

Total RNA was extracted from U87MG cells using the Direct-zol RNA MiniPrep (Zymo Research, Irvine, CA, USA) following manufacturer protocol. An on-column DNaseI treatment was included. RNA concentration and purity were determined by Nanodrop-1000 spectrophotometer and Qubit v.1 fluorometer plus Qubit RNA HS Assay Kit (Thermo Fisher Scientific, Wilmington, DE, USA).

0.5. µg of total RNA were retrotranscribed in 20μL using QuantiTect Reverse Transcription kit (Qiagen, Hilden, Germany) following manual protocol indications. Relative quantity of gene transcripts was measured by real-time PCR on samples’cDNA using a SYBRGreen chemistry and an iQ5 instrument (Bio-Rad, Milano, Italy). Two µL of 5-fold cDNA dilutions and 8 pmol of each oligonucleotide were added to 10 µL iTaq Universal SYBRGreen Supermix (Bio-Rad) in a 20 µL total reaction volume. The PCR cycle program consisted of an initial 30 s denaturation at 95 °C followed by 40 cycles of 5 s denaturation at 95 °C and 15 sec annealing/extension at 60 °C. A final melting protocol with ramping from 65 °C to 95 °C with 0.5 °C increments of 5 s was performed for verification of amplicon specificity and primer dimer formation. 

Primers were designed with Beacon Designer Software v.8.0 (Premier Biosoft International, Palo Alto, CA, USA) with a junction primer strategy, whenever possible. In any case, negative control of retro-transcription was performed to exclude any interference from residual genomic DNA contamination. The primers sequence for RealTime-PCR are reported in Table 1. 

### 4.5. Western Blot Analysis and MTT Cell Viability Assay

U87MG cells were seeded in 6-well plates (3 × 10^5^ cells/well) in a final volume of 2 mL/well and grown to 80% of confluence with standard medium (DMEM-high glucose). Cells were treated with vehicle (0.1% DMSO) or test compounds (i.e. 1 μM T1AM, SG-1, and SG-2) and incubated at 37 °C for 24h. After washing twice with ice-cold PBS cells were lysed and proteins (20–30 μg) were separated on Criterion TGXTM gel (4–20%) and transferred to Immuno-PVDF membrane (Bio-Rad). The membranes were probed overnight at 4 °C with primary antibodies [1:1000, SIRT6, m-TOR, p-mTOR, LC3, p62, and β-actin, (Cell Signaling), Euro Clone, Milan, Italy]. The primary antibodies were detected using peroxidase-coupled anti rabbit secondary antibody. The peroxidase was detected using a chemiluminescent substrate (ECL, Perkin Elmer, Milan, Italy). Immunoreactive bands were quantified performing a densitometric analysis with Image Lab Software (Bio-Rad) and normalized to β-actin. Cell viability was assessed by MTT reduction assay 24 h or 72h after SG-1, SG-2 or T1AM application (1 or 5 μM) to U87MG cells seeded at a density of 1 × 10^4^ cells per well of 96-well plates in 100 µL of culture medium. Briefly, the reaction was started by adding 10 μL of 3-(4,5-dimethylthiazol-2-yl)-2,5-diphenyltetrazolium bromide (MTT reagent; 5 mg/mL) to the medium and the cells were incubated for 3 h at 37 °C. The resultant insoluble MTT-formazan dark red crystals were dissolved with 100 μL of solubilization buffer (10% SDS in 0.01M HCl) and after overnight incubation at 37 °C absorbance at 570 nm was determined with a micro-plate reader (Bio-Rad 680).

### 4.6. Animals

Transgenic mhAPP mice (APP_Swe Ind_, line J20) overexpressing an alternatively spliced human APP (hAPP) minigene that encodes hAPP695, hAPP751, and hAPP770 bearing mutations linked to familial AD (V717F, K670N/M671L) have been used for electrophysiological assays. Transgenic mice and their littermate controls (C57BL/6J), minimum 3 months of age, were from a colony kept in the animal facility of the IN-CNR, Pisa, Italy).

All experiments were conducted according to the Italian Ministry of Health guidelines (Legislative Decree n. 116/92) and in accordance with the European Community guidelines (European Directive 86/609/EEC). The experimental protocol (IACUC document) was approved by the Ministry of Health (n. 192/2000-A).

### 4.7. In Vitro Electrophysiology

Electrophysiology was performed as previously described^19^. Briefly, mice (C57BL-6J mice, Jackson laboratory, Bar Harbor, ME, USA) were anesthetized with urethane (20% sol., 0.1 mL/100 g body weight) i.p. injections and then decapitated (Italian Ministry of Health, IACUC, December 13, 2000). Horizontal slices containing the entorhinal area were cut using a vibratome (VT1200S, Leica, Milano, Italy). All steps were performed in ice cold oxygenated ACSF (mM: 119 NaCl, 2.5 KCl, 2 CaCl_2_, 1.2 MgSO_4_, 1 NaH_2_PO_4_, 26.2 NaHCO_3_, 11 glucose). Slices were then transferred to a chamber and perfused at a 2–3 mL/min rate. Field potentials (FPs) were evoked by a concentric bipolar stimulating electrode in the layer II of EC. The recording pipette was placed in EC layer II. Basal recording was carried out using stimulus intensity evoking a response whose amplitude was 50–60% of the maximal amplitude. After 15 min of stable baseline, LTP was induced by high frequency stimulation (HFS, three trains of 100 pulses at 100 Hz, 10 s interval). After HFS, FPs were monitored every 20 s and averaged every 3 responses. The magnitude of LTP was calculated as the average of the relative amplitudes (compared to baseline) of FPs recorded in the last 10 min. Values were expressed as mean ± SEM percentage change relative to their mean baseline amplitude. Data collection and analysis were performed blindly by two different operators. SG2 compound (1 μM or 5 μM) was added to ACSF perfusion and administered to slices for 10 min, starting from 5 min before HFS. 

### 4.8. Statistical Analysis

All data are reported as mean ± SEM. Statistical analysis was performed by one-way analysis of variance (ANOVA), followed by Dunnett’s and Tukey’s multiple comparison tests. The threshold of statistical significance was set at *P* < 0.05. Data analysis was performed by Graph-Pad Prism 6.0 statistical program (GraphPad Software Inc., San Diego, CA, USA).

For electrophysiological experiments statistical comparisons between experimental groups or between FP amplitudes measured during baseline and after the induction protocol were performed by applying a one-way repeated-measures ANOVA with pair wise multiple comparison procedures (Holm–Sidak method, Sigmaplot 12.0, Systat Software, Inc., Chicago, IL, USA).

## Figures and Tables

**Table 1:** Primer sequences of target genes.

Reference Sequence (RefSeq) mRNA	Gene Symbol	Sense and Anti-Sense Sequences		Length of the Amplicon (bp)
NM_001243084	HIF1A	*sense*	TTGGCAGCAACGACACAG	169 bp
		*anti-sense*	GCAGGGTCAGCACTACTTC	
NM_004958	MTOR	*sense*	TGCCTTCACAGATACCCAGTA	171 bp
		*anti-sense*	AGACCTCACAGCCACAGA	
NM_000546	P53	*sense*	TCAACAAGATGTTTTGCCAACTG	118 bp
		*anti-sense*	ATGTGCTGTGACTGCTTGTAGATG	
NM_016539	SIRT6	*sense*	CTCCTCCGCTTCCTGGTC	119 bp
		*anti-sense*	TTACACTTGGCACATTCTTCC	
NM_002046	GAPDH	*sense*	CCCTTCATTGACCTCAACTACATG	115bp
		*anti-sense*	TGGGATTTCCATTGATGACAAGC	

Efficiency and specificity of primers were tested making standard curves with fivefold serial dilutions of a cDNA obtained from a pool of all samples. The first dilution was the two-fold diluted cDNA. All reactions were run in duplicate. All samples were analysed in duplicate and averaged. The relative expression of the target gene was normalized to the level of GAPDH in the same cDNA. Samples were analyzed by the 2^−ΔΔCt^ method, as described by Livak and Schmittgen [40].

## Supplementary Materials

The following are available online, Figure S1: Full-length blots relative to the cropped images showed in Figure 3.
