# Supplementary material for: Endogenous 3-Iodothyronamine (T1AM) and Synthetic Thyronamine-Like Analog SG-2 Act as Novel Pleiotropic Neuroprotective Agents through the Modulation of SIRT6
Source: Molecules. 2020 Feb 26;25(5):1054. doi: 10.3390/molecules25051054 (PMC7179148; doi:10.3390/molecules25051054)
Supplement: Supplementary file 1 [file molecules-25-01054-s001.pdf]

Endogenous 3-Iodothyronamine (T1AM) and synthetic thyronamine-like analog SG-2 act as novel pleiotropic neuroprotective agents through the modulation of SIRT6

Lorenza Bellusci[1#], Massimiliano Runfola[2#], Vittoria Carnicelli[1], Simona Sestito[1], Federica Fulceri[3], Filippo Santucci[4], Paola Lenzi[5], Francesco Fornai[5,6], Simona Rapposelli[2,7], Nicola Origlia[8], Riccardo Zucchi[1] and Grazia Chiellini[1*]

[1]Laboratory of Biochemistry, Department of Pathology, University of Pisa, Pisa, Italy; lorenza.bellusci@student.unisi.it (L.B.); vittoria.carnicelli@unipi.it (V.C.); simona.sestito@for.unipi.it (S.S.); riccardo.zucchi@med.unipi.it (R.Z.)
[2]Laboratory of Medicinal Chemistry, Department of Pharmacy, University of Pisa, Pisa, Italy; massimiliano.runfola@farm.unipi.it (M.R.); simona.rapposelli@unipi.it (S.R.)
[3]Department of Clinical and Experimental Medicine, University of Pisa, Pisa, Italy; federica.fulceri@unipi.it (F.F.)
[4]Sant'Anna School of Advanced Studies, Pisa, Italy; f.santucci@santannapisa.it (F.S.)
[5]Unit of Human Anatomy, Department of Translational Research and New Technologies in Medicine and Surgery, University of Pisa, Pisa, Italy; paola.lenzi@med.unipi.it (P.L.); francesco.fornai@med.unipi.it (F.F.)
[6]IRCCS Neuromed, Pozzilli, Italy (F.F.)
[7]Interdepartmental Research Centre of Ageing Biology and Pathology, University of Pisa, Pisa, Italy (S.R.)
[8]National Research Council (CNR), Institute of Neuroscience, Pisa, Italy; nicola.origlia@in.cnr.it (N.O.)
* Correspondence: grazia.chiellini@unipi.it (G.C.); Tel.: +39 050 2218662 (G.C.)
[#] L.B and M.R equally contributed

Supplementary Figures

Suppl. Figure 1

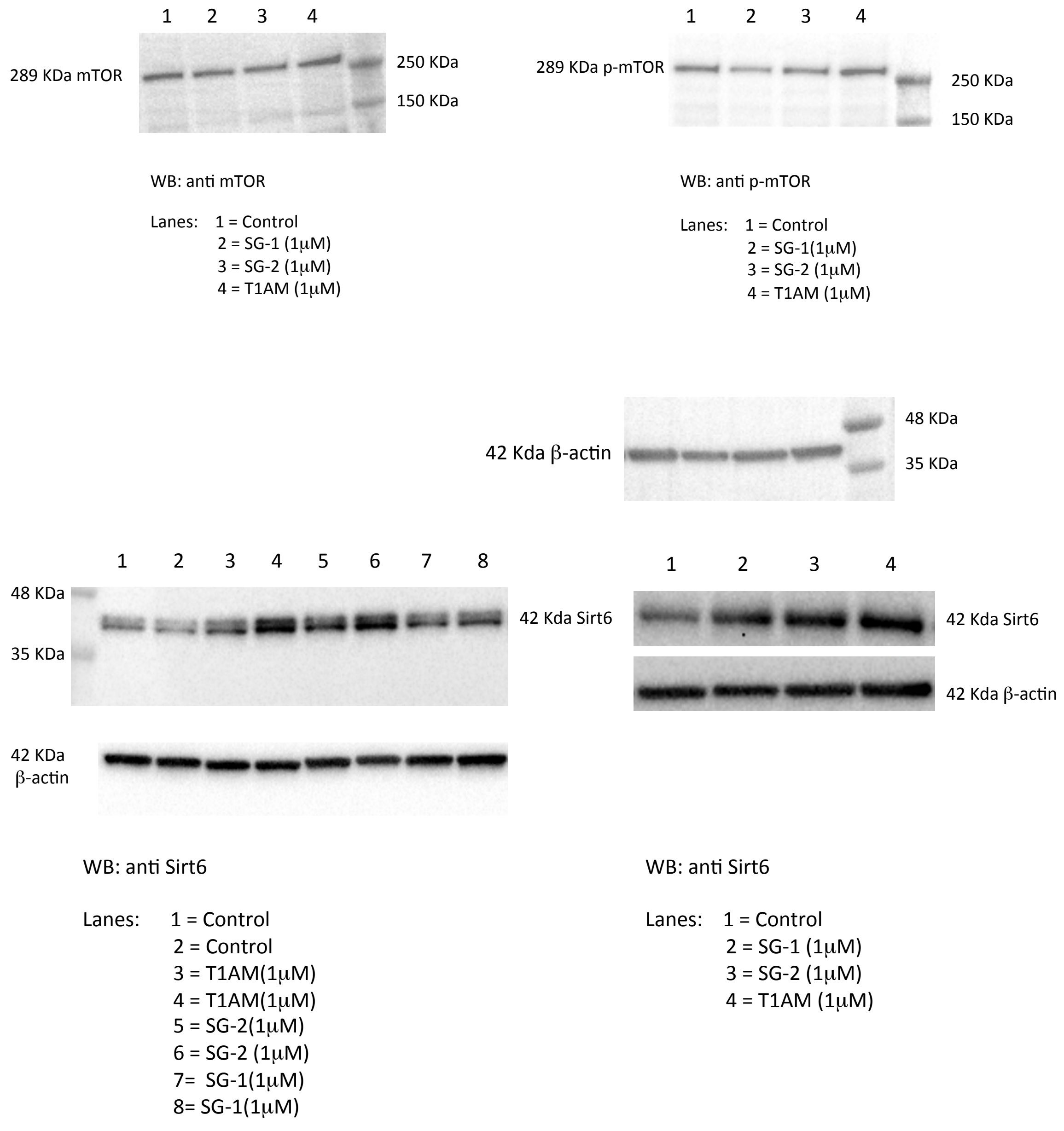

Supplementary Figure S1. Full-length blots relative to the cropped images showed in Figure 3. Uncropped Western blots relative to three different gels (n=3) shown in **Figure 3c** of the manuscript. The membranes were cut into sections to enable probing of the same blots with multiple antibodies. Therefore the above membrane sections are the full blots for each antibody.
